# Osteogenesis Imperfecta Due to Combined Heterozygous Mutations in Both *COL1A1* and *COL1A2*, Coexisting With Pituitary Stalk Interruption Syndrome

**DOI:** 10.3389/fendo.2019.00193

**Published:** 2019-03-28

**Authors:** Dongdong Wang, Mengmeng Zhang, Haixia Guan, Xiaoli Wang

**Affiliations:** ^1^Obstetrics and Gynecology Department of Shengjing Hospital, China Medical University, Shenyang, China; ^2^Department of Endocrinology and Metabolism, Institute of Endocrinology, Liaoning Provincial Key Laboratory of Endocrine Diseases, The First Affiliated Hospital of China Medical University, Shenyang, China

**Keywords:** pituitary stalk interruption syndrome, osteogenesis imperfecta, case report, next generation sequencing, skeletal malformation

## Abstract

Osteogenesis imperfecta (OI) is a hereditary connective tissue disorder, characterized by reduced bone content, fractures and skeletal malformation due to abnormal synthesis or dysfunction of type I collagen protein. Pituitary stalk interruption syndrome (PSIS) is usually associated with environmental and hereditary factors. Here, we report a rare case of OI and PSIS co-occurrence. A 19-year-old male patient sought treatment for growth delay and absent secondary sexual characteristics. Hormone measurements indicated the presence of hypopituitarism (secondary hypothyroidism, growth hormone deficiency, ACTH-cortisol hormone deficiency, hypogonadotropic hypogonadism). Pituitary magnetic resonance imaging indicated reduced morphology of the anterior lobe, absence of the pituitary stalk, and ectopic displacement of the posterior lobe to the infundibulum, supporting a diagnosis of PSIS. In addition, the patient, his monozygotic twin brother (no evidence of PSIS), and their mother all presented blue sclera and susceptibility to bone fractures before adulthood. Next-generation sequencing demonstrated that the family had compound heterozygous mutations in *COL1A1* and *COL1A2*, with no known mutations related to PSIS, pituitary hormone deficiency (PHD), or holoprosencephaly (HPE). The mother experienced breech and natural delivery of the patient and his brother, respectively. Thus, we deduced that the patient's PSIS might have resulted from breech delivery. Although we cannot exclude the possibility that the proband might have an undetected genetic abnormality causing PSIS or increasing his susceptibility to damage to the hypothalamic-pituitary region due to the limitation of exome sequencing, this rare case suggests that breech delivery in the newborn with OI might be related to PSIS.

## Background

Osteogenesis imperfecta (OI) is a group of inherited connective tissue disorders. It presents as reduced bone content and increased skeletal fragility, thereby increasing the likelihood of bone fractures (1). The etiology of most patients with autosomal hereditary OI (types I-IV) involves mutations in *COL1A1* (MIM 120150) and *COL1A2* (MIM 120160). These two genes encode the α1 and α2 chains of type I collagen, respectively. Together, two α1 chains and one α2 chain form the triple-helix structure of type I collagen. Heterozygous null mutations in *COL1A1* lead to haploinsufficiency and inadequate proα1 (I) production, thereby leading to type I OI. Type I OI has mild clinical symptoms and presents with an increased likelihood of bone fractures in childhood with or without blue sclera; in adulthood, the likelihood of bone fractures gradually decreases (1). Missense mutations in *COL1A1* or *COL1A2* usually lead to changes in collagen structure, thereby leading to clinical symptoms of moderate to severe type II-IV OI. Because new pathogenic genes that participate in the post-translational modification of type I collagen are continually being discovered, the number of OI types are also increasing continuously. Thus, studies have recommended reclassifying OI into 5 groups according to their pathogenic mechanism. OI caused by mutations in the *COL1A1* or *COL1A2* genes are classified under group A, namely, the group with defects in collagen synthesis, structure, or processing (1). Although many cases about OI are available in the literature, co-occurrence of OI with pituitary stalk interruption syndrome (PSIS) has never been reported.

Pituitary stalk interruption syndrome refers to a congenital abnormality of the anatomical structure of the pituitary. It has a typical presentation on magnetic resonance imaging, including a reduced or absent anterior pituitary, interruption, or loss of the pituitary stalk, and displacement of the posterior pituitary (2). Further, it leads to isolated or combined pituitary hormone deficiencies (3). Currently, its pathologic mechanism is still unknown. Pituitary stalk interruption syndrome is believed to be associated with hereditary and environmental factors, particularly perinatal events (3).

Here we report a family with OI that was caused by combined heterozygous mutations in both *COL1A1* and *COL1A2* genes. The proband showed co-occurrence of PSIS, whereas his older twin did not, suggesting that the proband either has a non-identified mutation or an epigenetic change related to environmental factors such as a perinatal event.

## Case Presentation

A 19-year-old male patient who presented with absent secondary sexual characteristics and delayed growth was admitted to the First Affiliated Hospital of China Medical University. The abnormalities in the patient were first noticed by his mother when she compared the patient with his monozygotic twin brother. During puberty, the gap in height between the patient and his twin brother was as much as 20 cm. The twins were born after a 37-week uneventful pregnancy. The mother experienced natural and breech delivery during her labor of the twin brother and the patient, respectively. Past medical history included one fracture on his right ulna when he was 2 years old. The patient's father was healthy. Both the patient's mother and his twin brother had blue sclera, and they both suffered from bone fractures multiple times before adulthood. The patient's parents stood at 172 cm (father) and 160 cm (mother). His twin brother was 170 cm tall.

### Physical Exam

**Figure 1:**
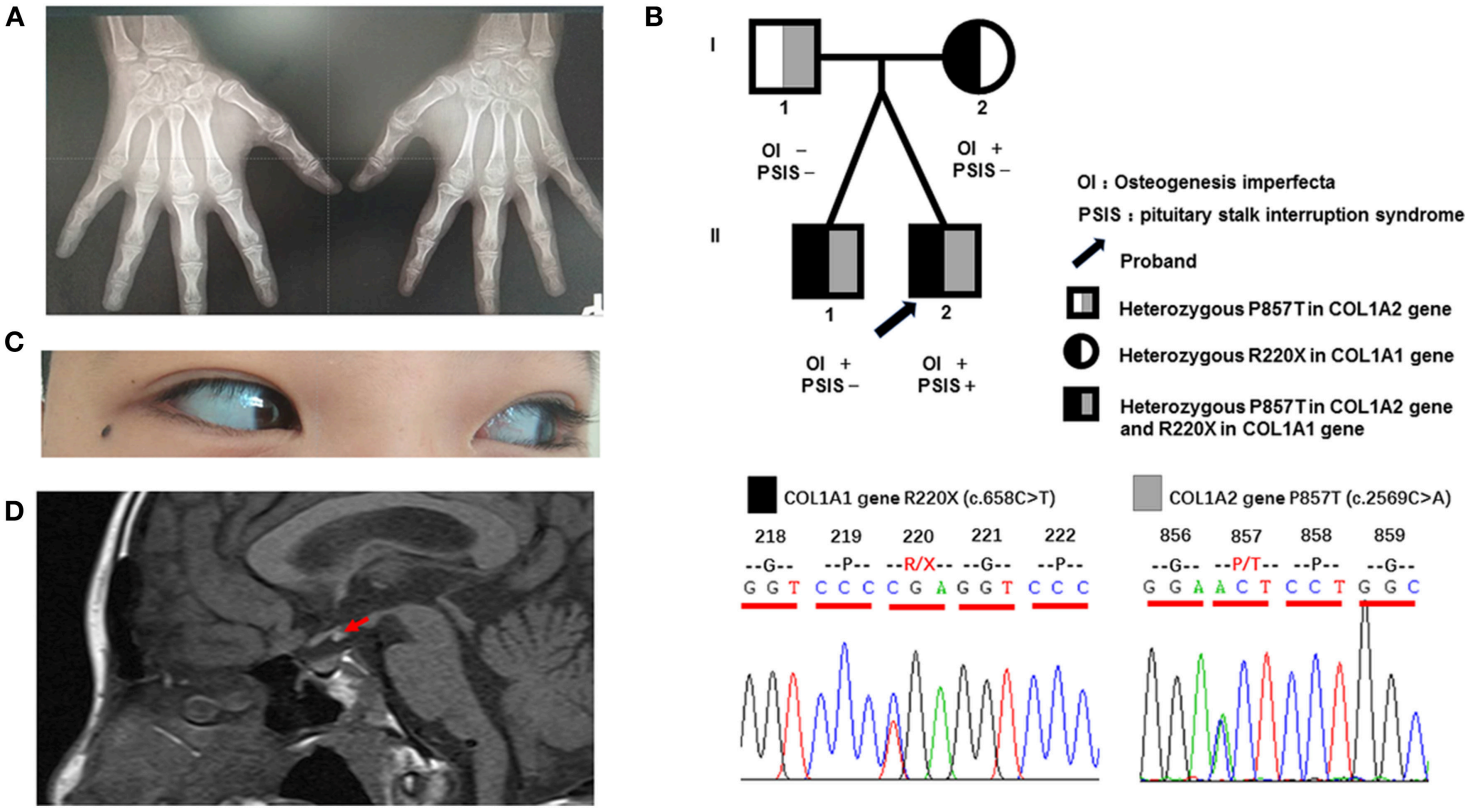
Clinical characteristics and gene sequencing results of the patient and his family members. **(A)** Digital radiography of the hands. The phalanges and humeral metaphysis are fused, indicating an age of 14–15 years. **(B)** Pedigree of the patient and gene sequencing results. The father is a heterozygous carrier of the P857T mutation in the *COL1A2* gene. The mother is a heterozygous carrier of the R220X mutation of the *COL1A1* gene. The twin brothers are heterozygous carriers of the above two mutations. **(C)** Light blue sclera of the patient. **(D)** Magnetic resonance imaging of the pituitary of the patient, indicating reduced morphology of the anterior lobe, absence of the pituitary stalk, and displacement of the neurohypophysis to the infundibulum (red arrow).

The patient showed the following features: height, 158 cm; weight, 53 kg; BMI, 20.70 kg/m^2^; arm span, 153 cm; upper body height, 67 cm; lower body height, 91 cm; triangular face; light blue sclera (Figure 1C); lack of facial hair; lack of Adam's apple; lack of underarm hair; lack of thyroid swelling; carinatum; enlarged breasts; palpable breast nodules; mild tenderness; lack of galactorrhea; lack of pubic hair; a stretched penis length of 4 cm. Both testes were palpable within the scrotum bilaterally.

### Work-Up

Testicular ultrasound showed that the right testis had a size of approximately 1.64 × 0.73 × 1.16 cm (0.73 ml), and the left testis had a size of ~1.48 × 0.65 × 1.01 cm (0.51 ml). His sense of smell was also tested formally (T & T olfactometer test), and no olfactory loss or hyposmia was found. Bone mineral density analysis showed decreased bone density in the lumbar vertebrae (Z = −3.1). Digital radiography of both hands indicated his skeletal age to be 14 years (Figure 1A). Magnetic resonance imaging of the pituitary gland showed reduced morphology of the anterior pituitary, absence of a pituitary stalk and ectopic displacement of the posterior lobe to the infundibulum (Figure 1D). These findings together suggested a diagnosis of PSIS. Ophthalmologic and audiologic examinations did not reveal any abnormalities.

Laboratory examinations demonstrated pituitary hypothyroidism, pituitary-adrenal axis dysfunction, hypogonadotropic hypogonadism, slightly increased prolactin level, and presence of insulin resistance with normal blood glucose level (Table 1). The basal GH level of the proband was < 0.5 μg/l and basal IGF-I level was < 25 ng/ml, suggesting a possibility of growth hormone deficiency, but we didn't perform stimulation tests because the patient and his mother refused to take the tests as well as be treated with growth hormone for economic reasons.

**Table 1:** Clinical and laboratory data of the patient at diagnosis and follow up.

	**At diagnosis**			**Follow up**			**Normal range**
				**1 month**	**3 months**	**6 months**	
Weight (Kg)	53			57	61	65	–
Hight (cm)	158			158	158.5	158.5	–
Volume of testis (ml)	0.51/0.73			–	–	1.47/1.69	–
T (nmol/l)	<0.69			4.85	4.54	4.64	9.08–55.23
FT (pmol/l)	2.14			67.25	69.39	65.04	55.05–183.5
LH (mIU/l)	<0.10			–	–	–	0.80–7.60
FSH (mIU/l)	0.59			–	–	–	0.70–11.10
TSH (mIU/l)	5.72			2.37	1.12	2.39	0.35–4.94
fT4 (pmol/l)	7.18			8.61	8.11	5.28	9.01–19.05
fT3 (pmol/l)	3.65			2.1	4.11	4.2	2.63–5.7
ACTH (pg/ml) (8:00 a.m.)	9.47			9.31	–	9.15	7.20–63.30
COR (nmol/l) (8:00 a.m.)	29.50			112.3	–	76.5	171–536
GH (μg/l)	<0.05			–	–	–	0.05–3.00
IGF-1 (ng/ml)	<25			–	–	–	115–358
PRL (mIU/l)	547.0			–	–	–	53–360
	Fasting	60 min after OGTT	120 min after OGTT	Fasting	Fasting	Fasting	
PG (mmol/l)	4.33	6.08	5.23	4.31	4.42	4.52	–
INS (mIU/l)	21.73	135.40	44.63	–	–	–	–
CP (pmol/l)	1621.10	7502.00	2579.00	–	–	–	–

*T, testosterone; FT, free testosterone; SHBG, sex-hormone binding globulin; LH, luteinizing hormone; FSH, follicle-stimulating hormone; TSH, thyrotropin-releasing hormone; fT4, free thyroxine; fT3, free triiodothyronine; ACTH, adrenocorticotropic hormone; COR, cortisol; GH, growth hormone; IGF-1, insulin like growth factor-1; PRL, prolactin; PG, plasma glucose; INS, serum insulin; CP, serum C peptide; OGTT, oral glucose tolerance test*.

### Genetic Testing

Blood samples were collected from the patient and his family members (father, mother, and twin brother). Genomic DNA was extracted using a blood extraction kit (Tian Jing Biochemical Technology Beijing, Ltd.). Roche Nimblegen SeqCap EZ Choice XL Library was used for exon trapping (including 4,132 genes). Illumina nextseq500 was used for high-throughput sequencing. Burrows-Wheeler Aligner software (BWA, 0.7.12-r1039) was applied to align the sequencing data to the human genome. ANNOVAR was used to annotate the genetic variants detected (based on the recent version of the dbSNP database, Clinvar, ExAC, and 1000 Genomes Project). Emphasis was laid on the analysis of known genes involved in OI, PSIS, PHD, and HPE according to pathology (listed separately in Table 2). The results revealed that the identical twins harbor heterozygous mutations in *COL1A1* (c.658C>T, p.Arg220Ter) and *COL1A2* (c.2569C>A, p.Pro857Thr). The mother harbors the same *COL1A1* mutation (p.Arg220Ter) and the father harbors the *COL1A2* (p.Pro857Thr) mutation (Figure 1B). No known pathogenic variants related to PSIS, PHD, or HPE were found in this family. No convincing discordant single nucleotide or indel variants were found in coding or regulatory regions in this phenotypically PSIS-discordant twin.

**Table 2:** List of genes related to bone metabolism, PSIS, PHD, and HPE.

**Official name**	**Phenotype**	**Inheritance**
**GENES RELATED TO BONE METABOLISM**		
COL1A1	Osteogenesis imperfecta, type I(a dominant form with blue sclerae);	AD
COL1A1, COL1A2	Osteogenesis imperfecta, type II(a perinatally lethal OI syndrome); Osteogenesis imperfecta, type III(progressively deforming form with normal sclerae); Osteogenesis imperfecta, type IV(a dominant form with normal sclerae)	AD
IFITM5	Osteogenesis imperfecta, type V	AD
SERPINF1	Osteogenesis imperfecta, type VI	AR
CRTAP	Osteogenesis imperfecta, type VII	AR
P3H1	Osteogenesis imperfecta, type XIII	AR
PPIB	Osteogenesis imperfecta, type IX	AR
SERPINH1	Osteogenesis imperfecta, type X	AR
FKBP10	Osteogenesis imperfecta, type XI	AR
SP7	Osteogenesis imperfecta, type XII	AR
BMP1	Osteogenesis imperfecta, type XIII	AR
TMEM38B	Osteogenesis imperfecta, type XIV	–
WNT1	Osteogenesis imperfecta, type XV	AR
IDS	mucopolysaccharidosis type II (MPS2; Hunter syndrome)	XLR
ARSB	Mucopolysaccharidosis type VI (Maroteaux-Lamy)	AR
SGSH	Mucopolysaccharidisis type IIIA (Sanfilippo A)	AR
NAGLU	Mucopolysaccharidosis type IIIB (Sanfilippo B)	AR
HGSNAT	Mucopolysaccharidosis type IIIC (Sanfilippo C)	AR
GNS	Mucopolysaccharidosis type IIID	AR
GALNS	Mucopolysaccharidosis IVA	AR
GLB1	Mucopolysaccharidosis IVB	AR
TNFRSF11B	Paget disease	AR
TNFRSF11A	Paget disease	AD
SQSTM1	Paget disease	AD
FGFR3	Achondroplasia	AD
ATP6VOA4	Renal tubular acidosis with deafness	–
ATP6V1B1	Renal tubular acidosis with deafness	AR
SLC4A1	Renal tubular acidosis with deafness	AD/AR
PHEX	Hypophosphatemic rickets, X-linked dominant	XLD
CLCN5	Hypophosphatemic rickets, X-linked recessive	XLR
FGF23	Hypophosphatemic rickets, autosomal dominant	AD
DMP1	Hypophosphatemic rickets, autosomal recessive, 1	AR
ENPP1	Hypophosphatemic rickets, autosomal recessive, 2	AR
SLC34A3	Hypophosphatemic rickets with hypercalciuria	AR
VDR	vitamin D-dependent rickets type 2A	AR
CYP27B1	Vitamin D-dependent rickets type 1A	AR
CYP2R1	Vitamin D-dependent rickets type 1B	AR
EHHADH	Fanconi renotubular syndrome autosomal dominant FRTS3	AD
SLC34A1	Fanconi renotubular syndrome autosomal recessive FRTS2	AR
**GENES RELATED TO PSIS**		
PROP1	Pituitary stalk interruption syndrome; Combined pituitary hormone deficiency	AR
LHX3	Pituitary stalk interruption syndrome; Combined pituitary hormone deficiency	AR
LHX4	Pituitary stalk interruption syndrome; Combined pituitary hormone deficiency	AD
OTX2	Pituitary stalk interruption syndrome; Combined pituitary hormone deficiency	AD
HESX1	Pituitary stalk interruption syndrome; Combined pituitary hormone deficiency	AR/AD
PROKR2	Pituitary stalk interruption syndrome; Kallmann syndrome	AD
GPR161	Pituitary stalk interruption syndrome; Combined pituitary hormone deficiency	–
CDON	Pituitary stalk interruption syndrome; Holoprosencephaly	AD
TGIF1	Pituitary stalk interruption syndrome; Holoprosencephaly	AD
**GENES RELATED TO PHD**		
BTK	Agammaglobulinemia and isolated hormone deficiency	XLR
POU1F1	Combined pituitary hormone deficiency	AR/AD
SOX3	Panhypopituitarism X-linked	XL
FGFR1	Hypogonadotropic hypogonadism with or without anosmia	AD
FGF8	Hypogonadotropic hypogonadism with or without anosmia	AD
*CHD7*	CHARGE syndrome	AD
**GENES RELATED TO HPE**		
SIX3	Holoprosencephaly	AD
SHH	Holoprosencephaly	AD
ZIC2	Holoprosencephaly	AD
PTCH1	Holoprosencephaly	AD
GLI2	Holoprosencephaly	AD
DISP1	Holoprosencephaly	–
FOXH1	Holoprosencephaly or cardiac defects	–
NODAL	Holoprosencephaly, Heterotaxy,visceral	AD
TDGF1	Holoprosencephaly, Forebrain defects	–
GAS1	regulator of SHH, Modifier of HPE	–
DLL1	Holoprosencephaly	–

*PSIS, Pituitary stalk interruption syndrome; PHD, pituitary hormone deficiency; HPE, holoprosencephaly*.

### Therapeutic Intervention and Follow-Up

For hypogonadism, 2000 U human chorionic gonadotropin (hCG) was given intramuscularly three times per week, and testosterone and free testosterone levels were re-examined periodically at outpatient visits. The underarm hair and pubic hair of the patient showed significant growth. The testicular volume of the patient increased to 1.47 ml (left) and 1.69 ml (right), and his testosterone levels increased as well but still not reach normal range (Table 1). Human menopausal gonadotropin (hMG) was added to the patient's medications (75 U along with hCG). For secondary hypothyroidism, levothyroxine administration was started at 25 μg once daily, thyroid function was monitored, and the dose was increased to 75 μg/day during follow-up. For secondary adrenal insufficiency, oral hydrocortisone was prescribed initially at 20 mg in the morning (8:00 a. m.) and 10 mg in the afternoon (3:00 p.m.), and the hydrocortisone dose was decreased to 5 mg/day during follow-up because the body weight of the patient increased quickly and he showed no manifestation of adrenal insufficiency. A dose of 15 mg hydrocortisone was recommended for the patient according to the guideline after carefully discussion with the patient and his family (4). For OI, bisphosphonates were recommended.

## Discussion

This paper reports a rare case of OI combined with PSIS; this patient was the index case in one OI family. In this family, the patient, his mother and twin brother presented blue sclera and bone fractures, whereas the father seemed clinically normal. Next-generation sequencing revealed that the twins harbor heterozygous mutations in both *COL1A1* and *COL1A2*. Currently, there are no reports on OI cases with such compound heterozygous mutations involving two genes. The *COL1A1* mutation (p.Arg220Ter) (rs72667036) was inherited from their mother. It causes termination of the α1 chain, thus resulting in a reduced amount of total type I collagen, which has been reported in many papers to result in type 1 OI (5–9). The *COL1A2* mutation (p.Pro857Thr) (rs150179964) was inherited from their father. It has an MAF < 0.01 in the Ensembl database, a SIFT score of 0.02 (deleterious), and a PolyPhen score of 0.036 (benign). A previous paper reported a Vietnamese type IV OI patient carrying p.Pro857Thr associated with a different mutation (p.Gly1012Ser) in *COL1A2* (10). The *COL1A2* mutation (p.Pro857Thr) was not located at the G (glycine) site of the G-X-X motif, which plays an important role in maintaining the normal protein structure of COL1A1 and COL1A2. Together with a lack of clinical presentation of OI in the patient's father, these results suggest that the *COL1A2* mutation (p.Pro857Thr) might have minor effects on the protein structure and function.

Osteogenesis imperfecta is a heterogeneous group of inherited connective tissue disorders that cause bone fragility and deformity (1). About 90% of OI cases are caused by structural or quantitative mutations in the collagen genes *COL1A1* and *COL1A2* (11, 12). In addition, alterations in more than 13 genes that are linked to collagen processing (*BMP1*), collagen modification (*CRTAP, LEPRE1/P3H1, PPIB, TMEM38B*), collagen folding and cross-linking (*SERPINH1, FKBP10, PLOD2*), bone mineralization (*IFITM5, SERPINF1*), and osteoblast development with collagen insufficiency (*SP7, WNT1, CREB3L1*) have been reported to account for OI. These genetic alterations provide further explanations for the pathogenesis, classification, and therapeutic targets of OI (1). Osteogenesis imperfecta is a major cause of skeletal deformities and may lead to secondary damages, such as hearing loss (13), dental abnormalities (14), neurological features including macrocephaly, hydrocephalus, syringomyelia, and basilar invagination (15), and short stature with a normal growth axis (16). Patients with OI may present multiple skeletal deformities, such as flat midface and triangular facies, scoliosis or kyphosis, and chest wall deformities including pectus excavatum, carinatum, and barrel chest (11).

Interestingly, in the present family, the twins shared the same genetic alterations that accounted for their OI, whereas the index case presented evident delay in growth and development. Obviously, OI was not the reason for these differences between the twins, which were attributed to PSIS. Pituitary stalk interruption syndrome is believed to be associated with genetic and environmental factors, especially perinatal events (3). More than 40 candidate genes for PSIS, PHD, and HPE have been reported (17–20), but only 5% of the PSIS patients were identified to harbor these defects (20, 21). Holoprosencephaly is characterized by severely disturbed midline brain development, and genetic studies have shown an association between PSIS and genes involved in HPE, suggesting that PSIS might be a mild form of HPE (20). Recent studies have suggested a polygenic etiology for isolated PSIS (19, 22, 23). Although there might be additional genetic and epigenetic alterations not yet recognized by the current sequencing technologies, it seems that environmental factors play more important roles in the pathogenesis of PSIS. As shown in previous studies, individuals who had a history of perinatal events, such as breech delivery, were more likely to develop PSIS (24, 25). The frequency of breech delivery among PSIS patients is much higher than that in the general population (26). The mechanisms behind the higher risk of PSIS caused by perinatal events remain unclear. Pituitary stalk interruption syndrome is reported to be associated with direct injury to the pituitary stalk, or transient anoxia of hypothalamus (3) during difficult labors. In our case of co-occurrence of OI and PSIS, next-generation sequencing did not show any abnormalities in genes related to PSIS, PHD, or HPE. Considering that the patient was born after a difficult breech delivery, and that his normally delivered twin brother did not present PSIS, we deduced that the PSIS might be a consequence of breech delivery. Studies assessing at-birth health outcomes of neonates with OI reported a high breech delivery rate (24–37%), suggesting that newborns with OI may benefit from additional clinical supervision (27, 28). However, we still cannot exclude the possibility that the proband may have an undetected genetic abnormality causing PSIS or increasing his susceptibility to damage to the hypothalamic-pituitary region, owing to the limitations of exome sequencing.

To the best of our knowledge, the available case reports on PSIS have never mentioned its co-occurrence with OI. The present report reminds us to further investigate the influence of OI in female patients on vaginal deliveries, perinatal events, and the incidence of PSIS among their offspring. If female OI patients unfortunately experience perinatal events, their offspring should be monitored for early identification of any pituitary dysfunction, which may lead to development and growth delay.

## Ethics Statement

The hospital ethics committee of China Medical University approved the study, and the patient and his family members provided written informed consent for publication of their clinical details and clinical images.

## Author Contributions

DW and XW: study design; MZ: data collection; DW and MZ: manuscript drafting; HG and XW: data interpreting and revision of the manuscript; DW, MZ, HG, and XW: approval of final version of the manuscript.

### Conflict of Interest Statement

The authors declare that the research was conducted in the absence of any commercial or financial relationships that could be construed as a potential conflict of interest.
